# A new mutation in the *RP1L1* gene in a patient with occult macular dystrophy associated with a depolarizing pattern of focal macular electroretinograms

**Published:** 2012-04-24

**Authors:** Takenori Kabuto, Hisatomo Takahashi, Yoko Goto-Fukuura, Tsutomu Igarashi, Masakazu Akahori, Shuhei Kameya, Takeshi Iwata, Atsushi Mizota, Kunihiko Yamaki, Yozo Miyake, Hiroshi Takahashi

**Affiliations:** 1 Department of Ophthalmology, Nippon Medical School Chiba Hokusoh Hospital, Chiba, Japan; 2 Department of Ophthalmology, Nippon Medical School, Tokyo, Japan; 3 Division of Molecular & Cellular Biology, National Institute of Sensory Organs, National Hospital Organization Tokyo Medical Center, Tokyo, Japan; 4 Department of Ophthalmology, Teikyo University School of Medicine, Tokyo, Japan; 5 Department of Ophthalmology, National Institute of Sensory Organs, National Hospital Organization Tokyo Medical Center, Tokyo, Japan; 6 Aichi Medical University, 21 Yazakokarimata, Nagakute-cho, Aichi, Japan

## Abstract

**Purpose:**

To determine whether a mutation in the RP1-like protein 1 (*RP1L1*) gene is present in a Japanese patient with sporadic occult macular dystrophy (OMD) and to examine the characteristics of focal macular electroretinograms (ERGs) of the patient with genetically identified OMD.

**Methods:**

An individual with OMD underwent detailed ophthalmic clinical evaluations including focal macular ERGs. Mutation screening of all coding regions and flanking intron sequences of the *RP1L1* gene were performed with DNA sequencing analysis in this case with OMD.

**Results:**

A new *RP1L1* mutation (c.3596 C>G in exon 4) was identified. The variant c.3596 C>G in exon 4 resulted in the substitution of cysteine for serine at amino acid position 1199. The serine at position 1199 is well conserved among the RP1L1 family in other species. Four out of five computational assessment tools predicted that this mutation is damaging to the protein function. This mutation was not present in 294 control alleles. The waveform of focal macular ERGs recorded from the patient with OMD had a depolarizing pattern, simulating the ERG waveforms observed after the hyperpolarizing bipolar cell activity is blocked.

**Conclusions:**

We have demonstrated in a Japanese patient the possibility that sporadic OMD may also be caused by an *RP1L1* mutation. The waveform of focal macular ERGs elicited from the OMD patient with the *RP1L1* mutation showed a depolarizing pattern. This characteristic is the same as reported for the focal macular ERGs of OMD.

## Introduction

Occult macular dystrophy (OMD; OMIM 613587) is an inherited macular dystrophy characterized by a progressive decrease in visual acuity with an essentially normal fundus and normal fluorescein angiograms [1,2]. The full-field electroretinograms (ERGs) are normal; however, the focal macular ERGs and multifocal ERGs (mfERGs) recorded from the macular area are abnormal [1-3]. Despite normal ophthalmoscopic findings, spectral domain-optical coherence tomography (SD-OCT) has shown morphological changes in the retina in the macular area [4-8]. Several studies have reported various degrees of disruption of the inner segment/outer segment (IS/OS) junction and the cone outer segment tip (COST) line [4-8].

The hereditary form of OMD is an autosomal dominant trait; however, sporadic patients have also been reported [3,9]. The gene responsible for the disease was recently identified as the RP1-like protein 1 (*RP1L1*) in four families with autosomal dominant OMD [10]. The *RP1L1* gene has been identified through sequence analyses of human and mouse genomes [11,12]. The human *RP1L1* gene is encoded in four exons that span 50 kb on chromosome 8p. The length of the mRNA of *RP1L1* is more than 7 kb, but the exact length varies among individuals because of the presence of several length polymorphisms. *RP1L1* encodes a protein with a minimal length of 2,400 amino acids and a predicted weight of 252 kDa.

The expression of RP1L1 is limited to the retina, and appears to be specific to photoreceptors [12]. The *RP1L1* gene was also found to be conserved in distant vertebrates [11]. Knockout mice lacking the RP1L1 protein have reduced ERG amplitudes and progressive photoreceptor degeneration [13]. The study of *RP1L1*^−/−^ mice also showed that the RP1L1 protein is located in the axoneme of the outer segments and connecting cilia exclusively in rod photoreceptors. The RP1L1 protein appears not to be expressed in cone photoreceptors in mice, although more than 97% of the photoreceptors in mice are rods [13]. However, immunohistochemical analysis of the RP1L1 of Cynomolgus monkeys with the human RP1L1 antibody showed that RP1L1 was expressed in rod and cone photoreceptors [10]. Because the amino acid sequence of human RP1L1 is only 39% identical to that of the mouse, researchers have suggested that the primate RP1L1 might have different functional roles in the cone photoreceptors of the retina than that of other species [10].

We have identified a new mutation in the *RP1L1* gene in a patient with clinical characteristics of OMD: abnormal focal macular ERGs and blurring of the IS/OS junction and the disappearance of the COST line in SD-OCT images. The fundus examination, fluorescein angiograms, and full-field ERGs were normal in this case. The mutation is an amino acid substitution of cysteine for serine in exon 4 of the *RP1L1* gene that has not been reported in the Single Nucleotide Polymorphism (SNP) database and was also not detected in any of the 294 normal control alleles. The serine at position 1199 is well conserved among the RP1L1 family in other species. Four out of five computational assessment tools (PolyPhen-2, SIFT, PMut, Align GVGD, and MutationTaster) predicted that this mutation is damaging to the protein function. A segregation of the mutation and the disease was found in one affected member and one unaffected member of the same family.

## Methods

The protocol conformed to the tenets of the Declaration of Helsinki and was approved by the Institutional Review Board of the Nippon Medical School and the ethics review committees of the National Hospital Organization Tokyo Medical Center. Written informed consent was obtained from all patients after the nature and possible consequences of the study were explained.

### Clinical studies

The ophthalmological examinations included best-corrected visual acuity (BCVA) measurements, refraction, slit-lamp biomicroscopy, ophthalmoscopy, fundus photography, perimetry, SD-OCT, fluorescein angiography (FA), full-field ERGs, focal ERGs, and mfERGs. The visual fields were determined with the Goldman perimetry and the Humphrey Visual Field Analyzer (model 745i; Carl Zeiss Meditec, Inc., Dublin, CA). The Swedish interactive threshold algorithm standard strategy was used with program 30–2 of the Humphrey Visual Field Analyzer. The OCT images were recorded using a SD-OCT (Carl Zeiss Meditec) on this patient and normal controls. Full-field scotopic and photopic ERGs were recorded using an extended testing protocol incorporating the International Society for Clinical Electrophysiology of Vision standards [14]. The full-field ERGs were used to assess retinal function under scotopic and photopic states.

### Focal macular electroretinograms

Focal macular ERGs were recorded with a commercial Focal Macular ERG system (ER80; Kowa Company, Tokyo, Japan, and PuREC; Mayo Company, Nagoya, Japan) using a bipolar contact lens electrode (MY type Electrode; Mayo Company). The stimulus and background lights were integrated into an infrared fundus camera [15-17]. The size of the stimulus spot was 15° in diameter and was placed on the macula by observing the infrared image of the retina on a monitor. The white stimulus and background illumination were generated by light-emitting diodes that had maximal spectral emissions at 440 to 460 nm and 550 to 580 nm, respectively. The luminances of the stimuli and background were 115.7 cd/m^2^ and 8.0 cd/m^2^. The duration of the stimulation was 100 ms. The responses were amplified and filtered with digital band pass filters from 5 to 200 Hz. Three hundred responses were summed with a stimulus frequency of 5 Hz. The a-wave, b-wave, d-wave, and oscillatory potentials (OPs) were evaluated.

### Multifocal electroretinograms

The mfERGs were recorded using a commercial mfERG system (LE-4000, Tomey, Nagoya, Japan; LE4100; Mayo Company, Inazawa, Japan). This system uses basically the same technology as the Visual Evoked Response Imaging System [18]. The visual stimuli consisted of 37 hexagonal elements with an overall subtense of approximately 50°. The luminance of each hexagon was independently modulated between black (2.47 cd/m^2^) and white (200.4 cd/m^2^) according to a binary m-sequence at 75 Hz. The surround luminance was set at 75.4 cd/m^2^.

### Mutation analysis

Blood samples were collected from the patient, and genomic DNA was isolated from peripheral white blood cells using a blood DNA isolation kit (NucleoSpin Blood XL; Macherey Nagel, Düren, Germany). The DNA was used as the template to amplify the *RP1L1* gene. Coding regions and flanking introns of the *RP1L1* gene were amplified with polymerase chain reaction (PCR) using primers produced by Greiner Bio-One (Tokyo, Japan). Primer sequences are listed in Table 1. The PCR products were purified (ExoSAP-IT; USB Corp., Cleveland, OH) and were used as the template for sequencing. Both strands were sequenced on an automated sequencer (Bio Matrix Research; Chiba, Japan). The identified mutations and coding polymorphisms were assayed in 294 control chromosomes from 147 healthy Japanese individuals with direct sequencing except the length polymorphism region. To sequence the length polymorphism region of the *RP1L1* gene, the amplified PCR products were subcloned into the StrataClone PCR cloning vector (Stratagene; La Jolla, CA). At least five cloned products from this case and 20 control individuals were sequenced on an automated sequencer.

**Table 1:** Sequences of oligonucleotide primers used in this study and pcr product size.

**Fragment name**	**Forward primer (5′-3′)**	**Reverse primer (5′-3′)**	**Product size (bp)**
RP1L1–2A	GAGACAGGAAATGCCAATCC	CCGCAACTGCTGAGCAGTGG	471
RP1L1–2B	CCTCTGCTCTGATAAGAAGC	TCCATGTGAGTATTTTGACC	373
RP1L1–3	CCTCCAGCTAGTGATAGAGG	GATTGACAGTACTGAGAAGG	498
RP1L1–4A	TTCCTTTATCCTGATGCTGC	CCAAAGACTTCCCTGCATCC	509
RP1L1–4B	TGTGGGAGGGCTACCCTTGG	GCTGACGAGTCCGAAGAAGC	508
RP1L1–4C	CTATGCATAGATGGAGCAGG	GTTACAGAGGAGTCCAGTGG	536
RP1L1–4D	CAATGTCCTCACCCAGCAGC	TCCAACCTGCAGAACCAAGG	494
RP1L1–4E	GACTCCTGCTCAAAATCTGG	GGACACCCTCTCCTGATTGG	784
RP1L1–4F	GGACAGCAGTCCCTGGAAGG	ACTGCACCGCCTCTTCTTGC	937
RP1L1–4G	AAACACAGTGCAAGAAGAGG	AGGCTCAAGCTGGGAGCCACTCTGC	variable
RP1L1–4H	GGGAAAGGCTCCCAGGAAGATGACC	TTCTGCACCTTCTGACTCTGGCTGG	1470
RP1L1–4I	CACAGAGGAACCCACAGAGC	GAGAAGGCCGAGAGGTTTCG	522
RP1L1–4J	CAAGAGAGAGCTCCAGAAGC	TCTGTTGAGTCTCTGGCTCC	547
RP1L1–4K	GACAAAGATCCCAAACTCGG	AGAGTCAGAAGATGTAGAGG	836
RP1L1–4L	TGAAGGGGAGATGCAAGAGG	GAGTGGGCCTGTCCTCAGGGACTGG	821
RP1L1–4M	AGGCTTCTGAAAGCAGCAGC	ACTATGGACATCTCCAGTGG	517

### Computational assessment of missense mutation

The effect of a missense mutation on the encoded protein was predicted with the PolyPhen-2, SIFT, PMut, Align GVGD, and MutationTaster online tools [19-24]. PolyPhen-2 is a software tool that predicts the possible impact of amino acid substitutions on the structure and function of human proteins using straightforward physical and evolutionary comparative considerations. SIFT generates multiple alignments of the sequence over different species to look at the conserved sequences of a gene; it assesses the conserved amino acid positions and analyzes the effect of missense changes on the conserved structure of proteins over the course of evolution. The SIFT tool assigns a score to the mutations, and a score of <0.05 is considered potentially damaging. PMut is software aimed at annotating and predicting pathological mutations. Align GVGD combines the biophysical characteristics of amino acids and protein multiple sequence alignments to predict where missense substitutions in genes of interest fall in a spectrum from enriched deleterious to enriched neutral. MutationTaster evaluates the disease-causing potential of sequence alterations.

### Statistical analysis

We calculated the 95% confidence intervals (CI) of the results of the focal macular ERGs of normal controls. There were 25 men and 21 women whose age ranged from 23 to 60 years (mean, 38.04±8.33 years) in this control group. We recorded focal macular ERGs from either of the eyes of normal controls and calculated the 95% CI of the amplitudes of the a-waves and the b-waves, the implicit time of the a-waves and b-waves, the potentials at 70 ms after the stimulus was turned on, and the time of the recovery of the b-wave to the baseline.

## Results

### Case report

**Figure 1:**
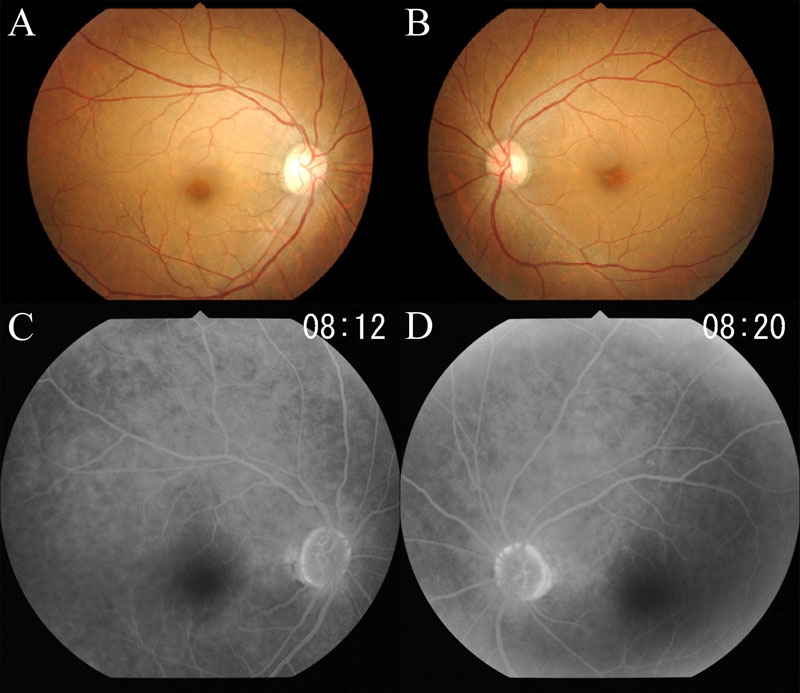
Fundus photographs (**A**, **B**) and fluorescein angiograms (**C**, **D**) of this case showing no abnormal findings.

**Figure 2:**
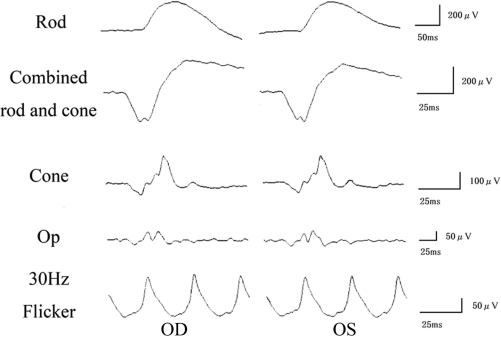
Full-field electroretinograms (ERGs) recorded according to the International Society for Clinical Electrophysiology of Vision (ISCEV) standards protocol in this case. The rod, combined rod-cone, cone, oscillatory potentials, and 30-Hz flicker full-field ERGs are shown. The results of full-field ERGs are within the normal limits in this case.

A 52-year-old woman complained of a gradual decrease in vision in both eyes during the past two to three years. Family history revealed no other members with any eye diseases, including her parents who were deceased. Her BCVAs were 20/63 in the right eye and 20/50 in the left eye. The fundus examination, fluorescein angiography, and full-field ERG results were within the normal limits (Figure 1A-D and Figure 2). The visual fields were full with the Goldman perimetry, but a relative central scotoma was detected in both eyes with the Humphrey Visual Field Analyzer.

### Spectral domain optical coherence tomography

**Figure 3:**
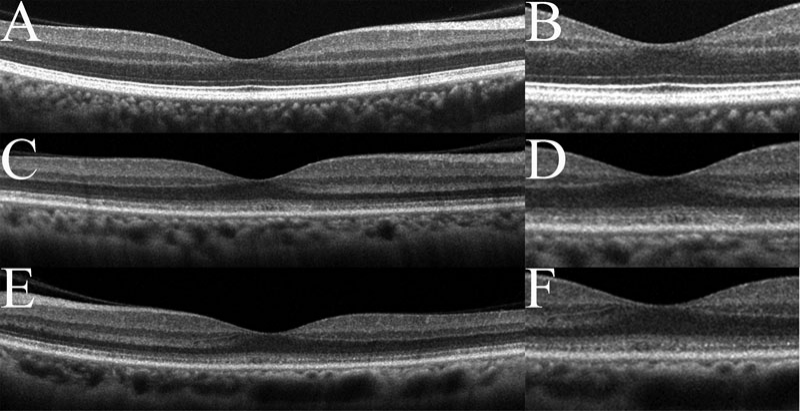
Spectral-domain optical coherence tomography (SD-OCT) findings of the eyes in normal controls (**A**, **B**) and in this case (**C-F**). Images from right eyes (**C**, **D**) and left eyes (**E, F**) are shown. Images at lower magnification (**A**, **C**, **E**) and higher magnification (**B**, **D**, **F**) are shown. The SD-OCT findings for the eyes in this case show obvious blurring of the IS/OS junction and the COST line. The COST line disappeared in the peripheral macula area in this case.

The SD-OCT images of this case showed a blurred IS/OS junction and COST line at the foveal center (Figure 3D,F). In the peripheral macula area, the COST line was absent, and only the blurred IS/OS junction was visible in this case (Figure 3C,E).

### Focal macular electroretinograms and multifocal electroretinograms

**Figure 4:**
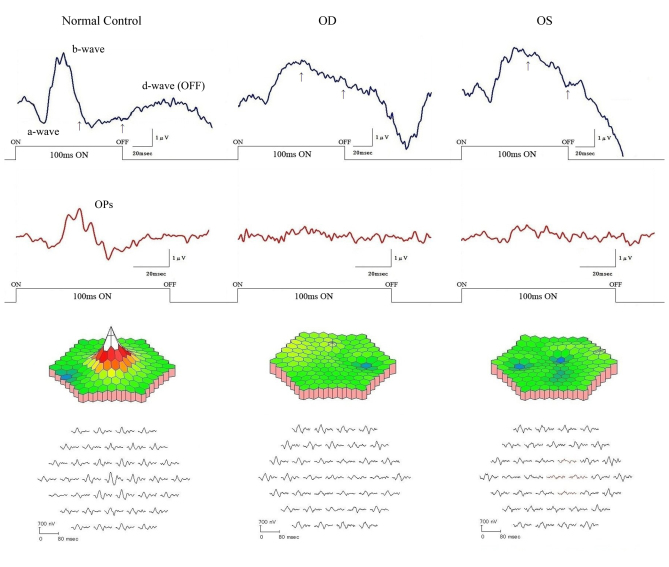
Results of focal macular electroretinograms (ERGs) and multifocal ERGs. Focal macular ERGs and oscillatory potentials recorded from a normal subject and this case are shown (top). The amplitude of the a-wave of this case was severely reduced, and the plateau region was significantly elevated (arrows). The topographic map and the local responses of multifocal ERGs recorded from the normal subject and this case are shown (bottom). The amplitudes in the foveal area were severely reduced in this case.

**Figure 5:**
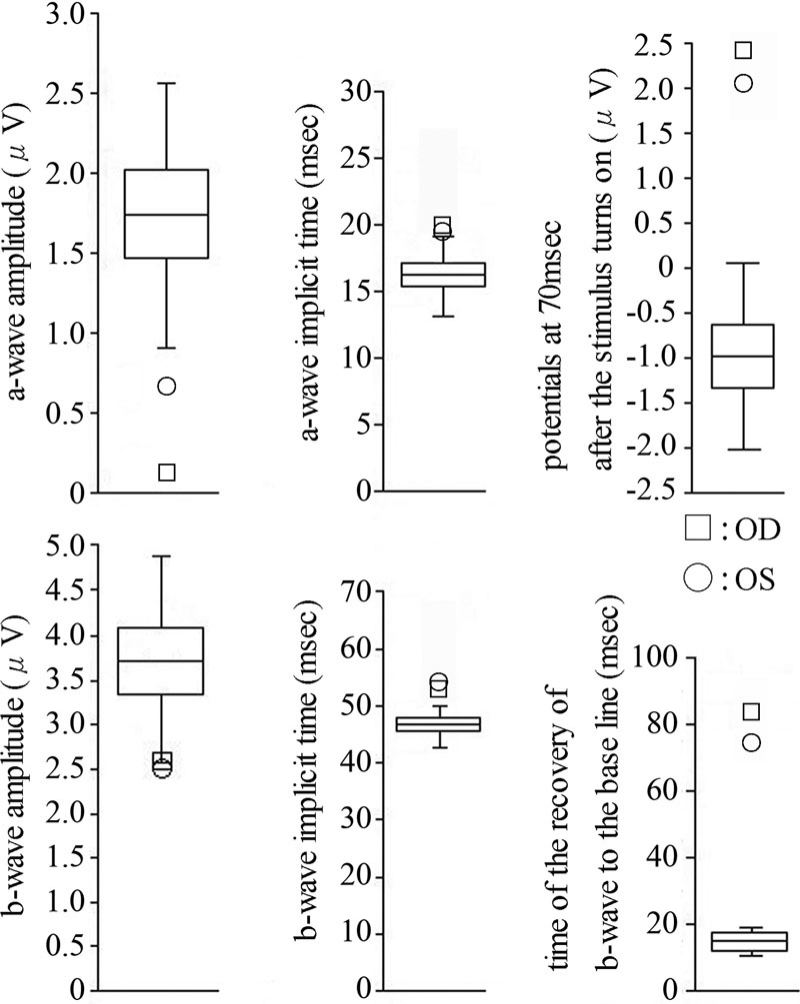
Plot of the amplitudes of the a-waves, b-waves, and the implicit time of the a-waves, b-waves, the potentials at 70ms after the stimulus turns on, and the time of the recovery of b-wave to the baseline for normal controls. There were 25 men and 21 women whose age ranged from 23 to 60 years (mean, 38.04±8.33 years) in this control group. The boxes represent the 95% confidence interval ranges, the horizontal line represents mean values, and the bars represent standard deviation. Data recorded from this case are plotted at indicated mark.

A severe reduction in the a-waves of the focal macular ERGs was found in this case (Figure 4). Although the b-waves were large, their shapes were abnormal. The b-waves rose to a peak, and the potential was maintained longer than normal. The plateau region of the b-wave was significantly elevated above the baseline potential (Figure 4, arrow). To analyze this characteristic, we quantified the potentials at 70 ms after the stimulus was turned on, and the recovery time of the descending slope of b-wave to the baseline from the peak of the b-wave. We calculated the 95% confidence intervals (CI) for the amplitudes of the a-waves and b-waves, the implicit times of the a-waves and b-waves, the potentials at 70 ms after the stimulus turns on, and the time of the recovery of the b-waves to the baseline obtained from the normal controls (Figure 5). Among these six parameters, the amplitudes of the a-waves, the implicit times of the b-waves, the potentials at 70 ms after the stimulus was turned on, and the time of the recovery of the descending slope of the b-wave to the baseline obtained from both eyes of this case were outside the range of the standard deviation and the 95% CI of the normal controls (Figure 5). Especially, the amplitudes of the a-waves, the potentials at 70 ms after the stimulus was turned on, and the time of the recovery of the descending slope of the b-wave to the baseline obtained from this case were severely affected. The amplitudes of the mfERGs in the foveal area were severely reduced in this case (Figure 4).

### Molecular genetic findings

**Figure 6:**
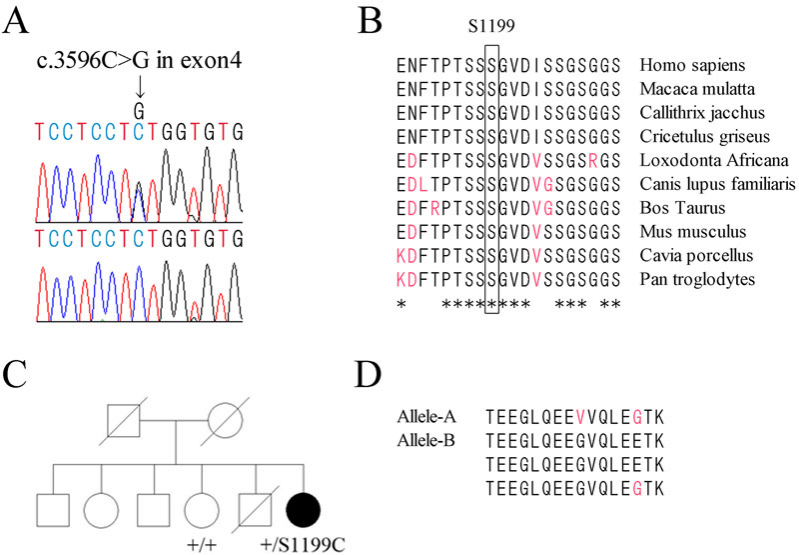
DNA analysis for c.3596C>G mutation and deduced amino acids of length polymorphism region of the RP1-like protein 1 (*RP1L1*) gene and the pedigree of the family with *RP1L1* gene mutation. **A**: Sequence chromatograms for this case (top) and the normal control (bottom) are shown. This case had a c.3596 C>G mutation in exon 4. **B**: Alignment of S1199 in the RP1L1 family proteins. Amino acid-sequence alignments of RP1L1 from 10 species reported in the NCBI database are shown. Amino acid residues of S1199 in humans and conserved residues from other species are boxed. The asterisks indicate completely conserved residues. S1199 is well conserved in all species reported. **C**: We confirmed that the mutation in Case 1 was segregated with the disease in one affected member and one unaffected member of the family. **D**: Deduced amino acids (AA) of repeated regions of the RP1L1 length polymorphism. In this case, one allele contains a 16 AA, and the other allele contains three 16 AA repeats. Variations of amino acids from reference sequence of RP1L1 are shown in red. Those variations are within normal limits.

Mutation analysis of the *RP1L1* gene in this case showed three missense mutations. There was a c.2578 C>T in exon 4 with a substitution of tryptophan (TGG) for arginine (CGG) at amino acid position 860, a c.3596 C>G in exon 4 with a substitution of cysteine (TGT) for serine (TCT) at amino acid position 1199, and a c. 4484 C>G in exon 4 with a substitution of arginine (CGC) for proline (CCC) at amino acid position 1495. The amino acid substitution at position 860 and 1495 has already been reported in the SNP database and is found in a high percentage of the normal population. A mutation at amino acid position 1199 has not been reported in the SNP database or in earlier reports (Figure 6A). The serine at position 1199 is well conserved among the RP1L1 family in other species (Figure 6B). This mutation was predicted to be probably damaging with a score of 0.999 by PolyPhen-2. The SIFT tool analysis revealed a score of 0 and predicted that the replaced amino acid is potentially damaging and would not be tolerated. PMut predicted that this mutation is pathological. Align GVGD predicted this mutation as class C65, which means it most likely interferes with the protein function. Out of five computational assessments, only MutationTaster predicted this mutation as a polymorphism. We confirmed that the mutation in this case was segregated with the disease in one affected member and one unaffected member of the family (Figure 6C). The unaffected member of the family in Case 1 underwent clinical examination, including BCVAs, slit-lamp biomicroscopy, fundus ophthalmoscopy, OCT, and focal ERGs. All examination findings were normal. This mutation was not present in 300 control alleles. This mutation p.S1199C has been registered in GenBank with accession number AB684329.

Bowne et al. [11] reported that *RP1L1* mRNA is variable due to the presence of a 48 bp polymorphic coding repeat. They reported that as many as six 48 bp repeats have been observed in normal controls. In this case, one allele contains a 48 bp repeat, and the other allele contains three 48 bp repeats (Figure 6D). There are variations of only two amino acids in the length polymorphism region from this case compared to the reference sequence (NP_849188). One variation with the substitution of E to G in the 14th amino acid of the length polymorphism region was in a previous report [12] (AAN86962, AAN86963, and AAN86964). The other variation with the substitution of G to V in the ninth amino acid of the length polymorphism region was found in more than 10 normal control alleles from a Japanese population. These variations of the length polymorphisms of *RP1L1* with one and three repeats have been registered in GenBank with accession numbers AB684331 and AB684332, respectively.

## Discussion

The mutation found in the *RP1L1* gene in this case was a missense mutation with cysteine substituted for serine at amino acid position 1199. This residue is well conserved among the RP1L1 family in other species, suggesting the importance of this amino acid residue for RP1L1 function. Four out of five computational analysis tools predicted this mutation is damaging to the protein function. We did not find this mutation in the sister of the patient with normal vision, although she was the only other family member we were able to test. To decide whether this mutation was pathogenic, we need to examine more family members and a larger number of normal controls. However, the phenotype of this case was typical of OMD, and thus the mutation in this case was most likely pathogenic.

The photoreceptor IS/OS junction and the COST line can be detected in the SD-OCT images of normal eyes [25-28]. Recently, several degrees of disruption of the IS/OS junction and/or COST line in the SD-OCT images of patients with OMD have been reported [4-8]. In our case, the IS/OS junction and the COST line appeared blurred in the SD-OCT images similar to previous reports.

Researchers have emphasized that the key to differentiating OMD from other diseases, such as optic neuritis or psychological disorders, is the recording of focal macular ERGs from the central retina [1-3]. Focal macular ERGs have a unique waveform when elicited by long-duration stimuli [29]. As shown in this patient, the waveform of focal macular ERGs recorded from patients with OMD with long-duration stimuli had a depolarizing pattern, simulating the ERG waveforms observed after the hyperpolarizing bipolar cell activity is blocked [30-33]. Researchers have demonstrated that by blocking hyperpolarizing bipolar cells with cis-2,3-piperidine dicarboxylic acid or kynurenic acid in monkeys, the a- and d-waves of photopic ERGs become smaller and the plateau between the b- and d-waves remains elevated above the baseline potential [34]. Full-field cone ERG in some human retinal dystrophies show a similar depolarizing pattern [29,35]. Kondo et al. [29] reported similar focal macular ERGs elicited with 100 ms stimuli from a patient with glittering crystalline deposits in the posterior fundus. The waveform of the focal macular ERGs of this case was similar to those reported for patients with OMD [31-33]. Because this case had a putative disease-causing mutation of the *RP1L1* gene, we suggest the reduced amplitude of the a-wave and the persistent plateau between the b- and d-waves of the focal macular ERGs elicited with long-duration stimuli might be specific markers that could help diagnose OMD.
